# Inherited CD19 Deficiency Does Not Impair Plasma Cell Formation or Response to CXCL12

**DOI:** 10.1007/s10875-023-01511-w

**Published:** 2023-05-29

**Authors:** Kieran Walker, Anoop Mistry, Christopher M. Watson, Fatima Nadat, Eleanor O’Callaghan, Matthew Care, Laura A. Crinnion, Gururaj Arumugakani, David T. Bonthron, Clive Carter, Gina M. Doody, Sinisa Savic

**Affiliations:** 1 Leeds Institute of Medical Research, University of Leeds, St. James’s University Hospital, Beckett Street, Leeds, LS9 7TF UK; 2 Department of Clinical Immunology and Allergy, St James’s University Hospital, 5.18 Clinical Sciences Building, Beckett Street, Leeds, LS9 7TF UK; 3 Yorkshire and North East Genomic Laboratory Hub, Central Lab, St. James’s University Hospital, Leeds, LS9 7TF UK; 4 Department of Clinical Genetics, Chapel Allerton Hospital, Leeds, LS7 4SA UK; 5 National Institute for Health Research, Leeds Biomedical Research Centre and Leeds Institute of Rheumatic and Musculoskeletal Medicine (LIRMM), St James’s University Hospital, Leeds, LS9 7TF UK

**Keywords:** CD19, plasma cells, antibody deficiency, CXCR4

## Abstract

**Background:**

The human CD19 antigen is expressed throughout B cell ontogeny with the exception of neoplastic plasma cells and a subset of normal plasma cells. CD19 plays a role in propagating signals from the B cell receptor and other receptors such as CXCR4 in mature B cells. Studies of CD19-deficient patients have confirmed its function during the initial stages of B cell activation and the production of memory B cells; however, its role in the later stages of B cell differentiation is unclear.

**Objective:**

Using B cells from a newly identified CD19-deficient individual, we investigated the role of CD19 in the generation and function of plasma cells using an in vitro differentiation model.

**Methods:**

Flow cytometry and long-read nanopore sequencing using locus-specific long-range amplification products were used to screen a patient with suspected primary immunodeficiency. Purified B cells from the patient and healthy controls were activated with CD40L, IL-21, IL-2, and anti-Ig, then transferred to different cytokine conditions to induce plasma cell differentiation. Subsequently, the cells were stimulated with CXCL12 to induce signalling through CXCR4. Phosphorylation of key downstream proteins including ERK and AKT was assessed by Western blotting. RNA-seq was also performed on in vitro differentiating cells.

**Results:**

Long-read nanopore sequencing identified the homozygous pathogenic mutation c.622del (p.Ser208Profs*19) which was corroborated by the lack of CD19 cell surface staining. CD19-deficient B cells that are predominantly naïve generate phenotypically normal plasma cells with expected patterns of differentiation-associated genes and normal levels of CXCR4. Differentiated CD19-deficient cells were capable of responding to CXCL12; however, plasma cells derived from naïve B cells, both CD19-deficient and sufficient, had relatively diminished signaling compared to those generated from total B cells. Additionally, CD19 ligation on normal plasma cells results in AKT phosphorylation.

**Conclusion:**

CD19 is not required for generation of antibody-secreting cells or the responses of these populations to CXCL12, but may alter the response other ligands that require CD19 potentially affecting localization, proliferation, or survival. The observed hypogammaglobulinemia in CD19-deficient individuals is therefore likely attributable to the lack of memory B cells.

## Introduction

Antigen dependent B cell activation and B cell development is largely regulated by signals received through the B cell antigen receptor (BCR) and cell surface molecules such as CD19 and CD21 [1–3]. Together these make up the BCR complex. BCR activation results in a cascade of molecular events including the activation of the spleen tyrosine kinase SYK, phosphorylation of tyrosine residues in the cytoplasmic tail of CD19 and the activation of phosphatidylinositol-3- kinase (PI3K)/AKT and ERK pathways [4]. Several lines of evidence highlight the importance of CD19 in BCR signalling. Firstly, disruption of the actin cytoskeleton alone has been shown to activate BCR signalling in a CD19-dependent manner [5, 6]. Secondly, stimulation of CD19 lowers the threshold needed for activation of B cells by several orders of magnitude [2, 7]. Finally, the CD19/CD21 complex has been shown to prolong BCR signalling by stabilizing the BCR in plasma membrane lipid rafts and blocking internalization of the BCR [8]. Moreover, the BCR complex is thought to integrate signals from multiple other receptors including toll-like receptors, CD40, BAFFR and chemokine receptors, all of which depend on CD19 for signal propagation [9–13].

Murine and human models indicate that loss of CD19 results in an overall decrease in the humoral response and an increased susceptibility to bacterial infection [14–16]. To date 10 patients with CD19 deficiency causing an absence of CD19 on the cell surface have been reported [17–22]. All patients had a low IgG level and recurrent infections of the respiratory tract. Other features included recurrent bacterial conjunctivitis, meningitis, and gastroenteritis [16].

The affected individuals all had a reduced proportion of class switched memory B cells suggesting a defect in early memory B cell formation. In keeping with this, experimental data show that CD19 deficiency leads to impaired somatic hypermutation and a reduction in the production of class-switched immunoglobulins after ex vivo stimulation; in contrast, IgM secretion was intact, suggesting that the generation short-lived antibody-secreting cells was not affected [19, 21–23]. However, several lines of evidence indicate that CD19 expression may effect plasma cell development and function. For example, a small proportion of long-lived bone marrow plasma cells downregulate the expression of CD19. These cells show an increased frequency of V gene somatic mutation, whereas there is an absence of CD19- plasma cells in the bone marrow of infants [24, 25]. The plasma cell compartment in CD19 deficiency patients is yet to be fully described. Here we describe a patient with genetically proven CD19 deficiency and investigated the role of CD19 in the generation and function of plasma cells using an in vitro differentiation model.

## Methods

### Patient Cohort

The patient, family members and healthy donor volunteers provided written informed consent to participate in this study. Ethical approval was granted by the Leeds East Research Ethics Committee (18/YH/0070 and 07/Q1206/47).

### Flow Cytometry

Lymphocyte phenotyping, representing our standard clinical panel, was performed to determine the percentage and absolute number of CD3^+^, CD4^+^, and CD8^+^ T cells, CD19^+^ B cells, and CD3^-^ CD56^+^ NK cells. In brief, whole blood was added to flourochrome labelled antibodies that recognized cell surface determinants on T, B and NK cells (CD45/CD3/CD4/CD8/CD19/CD16-56) (Becton Dickinson, Berkshire, UK). Following erythrocyte lysis, the samples were acquired on a FACSCanto^TM^ II flow cytometer using BD FACSCanto^TM^ Clinical Software v.3 (Becton Dickinson). TruCount^TM^ tubes (Becton Dickinson) were used to determine the percentage and absolute value of the subsets measured. Additional immunophenotyping of B cells was performed using the following antibodies: CD20-APC (Clone L27; BD Biosciences), CD20-BV421 (Clone L27; BD Biosciences), CD19-BV421 (Clone HIB19; BD Biosciences), CD19-APC (Clone SJ25C1; eBioscience), CD19-PE (Clone LT19; Miltenyi), CD81-FITC (Clone JS-81; BD Biosciences), CD27-PE (Clone M-T271; BD Biosciences), IgD-V500 (Clone IA6-Z; BD Biosciences), CD138-APC (Clone 44F9; Miltenyi) and CD21 (Clone B-ly4; BD Biosciences). Cells used in the differentiation protocol were stained in FACs buffer (PBS, 0.5% BSA, 0.05% sodium azide) with anti-CD20-V450 (Clone 2H7; Thermofisher), anti-CD19-PE (Clone LT19; Miltenyi), anti-CD27-FITC (Clone M-T271; BD Biosciences), anti-CD38-PECy7 (Clone HB-7; BD Biosciences), anti-CD138-APC (Clone 44F9; Miltenyi), 7-AAD (BD Parmingen) and anti-CXCR4-PE (Clone 12G5; R&D Systems). Cells were first gated on their forward vs side scatter characteristics, followed by doublet exclusion using forward scatter area against forward scatter height and then live-dead discrimination using 7-AAD. Cell number was assessed using CountBright™ beads (Thermofisher). Samples were run on a Beckman Coulter Cytoflex S flow cytometer and analyzed using FlowJo version 10 (BD Biosciences) and Prism 8 (GraphPad).

### Nanopore Sequencing

Following written consent, DNA was isolated from peripheral blood lymphocytes of the proband and her relatives, using a Chemagic 360 automated extractor (Perkin Elmer, Waltham, MA, USA). A 7,476-bp long-range PCR amplicon was optimised to amplify the *CD19* coding sequence using primers dAGTGTTGTGAGTCTGGAGGG (forward) and dCTGGAAGTGTCACTGGCATG (reverse). Amplification products were resolved on a 1% tris-borate EDTA agarose gel then purified using a QIAquick column, eluting in 30 μL of buffer EB (Qiagen GmbH, Hilden, Germany). Nanopore sequencing was undertaken on the gel-purified amplification products. An end-repair and nickase treatment reaction was first performed then sequencing adapters were ligated to the double-stranded DNA. A 24-hr Flongle sequencing run was initiated using MinKNOW software v.3.6.5 (ONT).

Offline basecalling converted raw data from fast5 to FASTQ format using Guppy v.3.6.0 (http://nanoporetech.com). Adapter sequences were trimmed from the resulting reads using Porechop v.0.2.3 (https://github.com/rrwick/Porechop) before NanoFilt v.2.2.0 was used to remove low quality reads (Q ≤6) (https://github.com/wdecoster/nanofilt) [26]. Sequence reads were then aligned to an indexed human reference genome (build hg19) using minimap2 v.2.16 (https://github.com/lh3/minimap2) [27] before being sorted by alignment coordinate and calling was performed at the *CD19* locus using NanoPolish v.0.13.2 (https://nanopolish.readthedocs.io). Each variant was annotated using Alamut Batch standalone (v.1.11; database version 2020.03.18) (Interactive Biosoftware, Rouen France) before its clinical significance was assessed according to Association for Clinical Genomic Science best practice guidelines [28]. Aligned sequence reads were manually inspected using the Integrative Genomics Viewer v.2.4.10 (http://software.broadinstitute.org/software/igv/) [29]. Summary read metrics were generated using NanoStat v.1.1.2 (https://github.com/wdecoster/nanostat) [26].

To confirm and genotype the putative pathogenic *CD19* variant, a 436 bp amplicon was designed and optimised before being Sanger sequenced using an ABI3730, following manufacturer’s protocols (Applied Biosystems, Paisley, UK). Sequence chromatograms were visualised using 4Peaks v.1.8 (http://www.nucleobytes.com).

### B Cell Purification and In Vitro Differentiation

Peripheral blood was obtained from healthy donors and the affected patient after informed consent. Mononuclear cells were obtained following lymphoprep density centrifugation. B cell selection was carried out with a Miltenyi memory B-cell isolation kit, selecting for untouched total B cells using the first step of the manufacturer’s protocol which depletes non-B cells. Alternatively, naïve B cells were enriched by depletion of memory cells using anti-CD27 MACs beads (Mitenyi). B-cells were cultured as previously described [30, 31] in 24-well plates at 1×10^5^ cells/ml in IMDM + 10% FBS with MEM amino acid solution (1:50) and Lipid Mixture 1, chemically defined (1:200) (all Thermo Fisher Scientific) and the addition of 20 U/ml human IL-2 (Roche), 50 ng/ml human IL-21 (Peprotech) and 10 μg/ml F(ab′)_2_ goat anti-human IgM/IgG (Jackson Immunoresearch) on previously irradiated CD40L-expressing fibroblasts. After 3 days, B cells were removed from the CD40L-cells by gentle pipette mixing. B cells were reseeded at in media containing 20 U/ml human IL-2 and 50 ng/ml human IL-21. On day 6, cells were seeded in media containing human IL-6 (10 ng/ml), human IL-21 (50 ng/ml), 100 U/ml multimeric APRIL (Adipogen), and 100nM gamma secretase inhibitor (GSI) inhibitor (L-685458; Sigma) and re-fed at day 10.

### Antibody Analysis

A multiplex flow immunoassay to measure anti-nuclear antibodies (ANA) including specificities dsDNA, Ro60, Ro52, La, Sm, SmRNP, RNP-68, CENP-B (centromere), Scl-70, Jo-1, chromatin and ribosomal P Ab was performed using a Bioplex 2200 (Bio-Rad). ELISAs were performed with Human IgM ELISA Quantitation Set (E80-100) or Human IgG ELISA Quantitation Set (E80-104) (Bethyl Laboratories Inc) according to the manufacturer’s instructions. ELISA absorbance values were analyzed at 450 nm and Ig concentrations calculated from standard curves.

### Gene Expression Analysis

RNA was purified using TRIzol (Invitrogen). Library preparation and 150-bp paired-end sequencing on the NovaSeq6000 (Illumina) were performed by Novogene. Fastq files from the sequencing runs were subject to initial quality assessment, trimming, alignment, and annotation. Transcript abundance was estimated using RSEM v1.3.0 and processed using DESeq2. Analysis of differential exon usage of CD19 transcripts was performed using DEXSeq (v.1.30). Exon usage and splicing were additionally visualized using IgV. Expression datasets are available with Gene Expression Omnibus accession numbers GSE222861 and GSE219012.

### B Cell Stimulations and Protein Analysis

Anti-CD19 beads were prepared by pre-coupling biotinylated αCD19 (clone HD37, Absolute Antibody) to anti-biotin MACSiBead particles (Miltenyi) as per manufacturer’s instructions for the T cell activation/expansion kit. Prior to use the antibody-bead mix was resuspended in the same media as the B cell cultures. At day 6 and day 13 cells were washed, counted, and re-suspended at 1–2×10^6^ in 0.5% serum IMDM containing IL-6/IL-21/APRIL. Cells were incubated for 12–20 h at 37°C. Following this, cells were placed in a 37°C water bath. An unstimulated sample of cells was taken prior to the addition of either 1ng/ml CXCL12 (Peprotech) or αCD19 stimulating beads. Samples were then taken after 1, 5, and 10 min post stimulation.

Protein lysates were separated on an SDS-polyacrylamide gel and transferred to nitrocellulose membrane. The membranes were probed with antibodies to phospho-AKT (T308), phospho-AKT (S473), pan AKT, phospho-ERK or pan ERK (all Cell Signaling) and then incubated with an HRP-coupled secondary antibody (Jackson Immunoresearch). HRP was then detected with ECL substrate (Thermofisher). The signal was visualized using a Bio-Rad ChemiDoc^TM^ MP Imaging System, which uses ImageLab^TM^ software for protein quantification or alternatively quantified using ImageJ following exposure to film (Amersham Hyperfilm ECL; GE Healthcare) developed using an X-ray developer (SRX-101A).

## Results

### Case Description

A 30-year-old female born to healthy related parents presented with 3 episodes of radiologically confirmed pneumonia and one episode of *Streptococcus pneumoniae* septicaemia. Additionally, she had an isolated episode of *Campylobacte*r gastroenteritis and a long-standing history of recurrent bacterial ear and sinopulmonary infections (Table 1).

**Table 1:** Clinical manifestation features of patient with novel CD19 variant

Age	Infection	Investigations	Treatment
30	Chest infection requiring hospital admission	No organisms isolated, normal chest X ray	48 h of intravenous amoxicillin + potassium clavulanate and clarithromycin. Followed by 12 days of oral equivalent.
28	Community acquired pneumonia followed by hospital acquired pneumonia	Confirmed chest X ray changes with left lower zone consolidation, required 2-week hospital stay including iv antibiotics. No organisms isolated. Subsequent readmission with new chest X-ray changes, treated as hospital acquired pneumonia	Initially intravenous ceftriaxone followed by oral course. Subsequently changed to amoxicillin and clarithromycin intravenously for 48 hours and then a 7-day course of oral regime of the same antibiotics Patient was offered oral phenoxymethylpenicillin prophylaxis after this admission.
27	Community acquired pneumonia	Consolidation in right upper zone on chest radiograph Penicillin sensitive *streptococcus pneumonia* positive blood culture	Oral amoxicillin for 7 days
26	*Varicella Zoster* reactivation		Oral acyclovir for 7 days
24-25	Recurrent episodes of Otitis Media		Repeated courses of amoxicillin and clarithromycin
23	Community acquired pneumonia	Right mid zone consolidation on chest radiograph	Oral amoxicillin for 10 days
20	Community acquired pneumonia	Left perihilar consolidation on chest radiograph Penicillin sensitive *streptococcus pneumonia* positive blood culture	Intravenous benzylpenicillin followed by oral amoxicillin
10	Gastroenteritis	*Camylobacter* positive stool culture.	

**Fig. 1:**
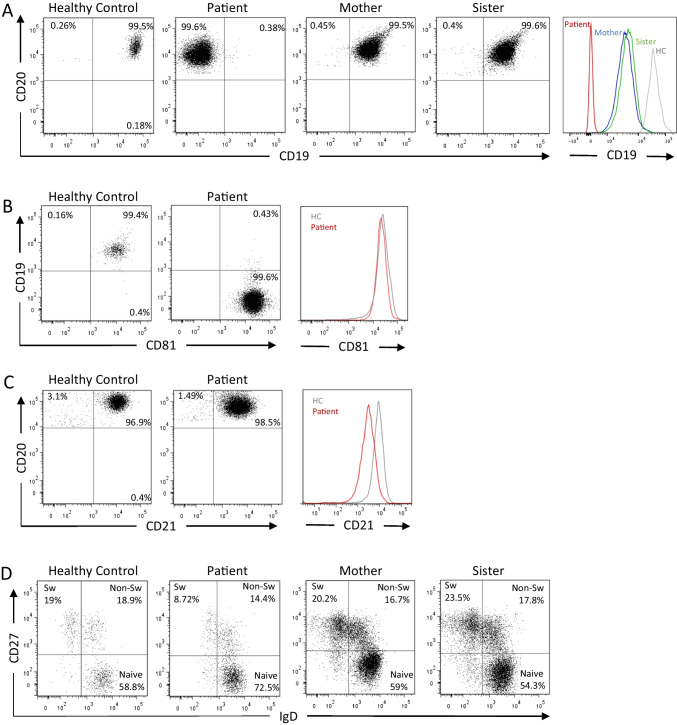
Loss of CD19 surface expression and reduced memory B cells. The peripheral blood B cells from a healthy control, the patient, her mother (A, D) and sister (A, D) were evaluated by flow cytometry using antibodies against (A) CD20 and CD19 (B) CD19 and CD81 (C) CD20 and CD21 or (D) CD27 and IgD. Sw, switched memory B cells. Non-Sw, non-switched memory B cells

Immunological investigations showed a low polyclonal immunoglobulin G (3.5 g/l, reference range 6–16), with a normal level of IgA and IgM and no detectable autoantibodies. She made a poor response to pneumococcal polysaccharide vaccination, in keeping with her clinical picture. Initial lymphocyte profiling showed normal numbers of T and NK cells and an apparent lack of B cells as assessed by CD19 expression (Table 2). Further flow cytometric evaluation confirmed the presence of a polyclonal CD20+CD81+CD21+CD19- B cell population using three different monoclonal CD19 antibodies, with reduced levels of CD21 (HC n=17 MFI 11009 ±1489; patient MFI 5020) and a reduction in memory B cell populations, in contrast to unaffected family members who exhibited relatively normal B cell phenotypes apart from an apparent reduction in surface CD19, similar to other affected families [17] (Fig. 1 A, B, C, D, and Table 2).

**Table 2:** Percentage of CD3^+^, CD4^+^, and CD8^+^ T cells, CD3^-^CD56^+^ NK cells, CD19^+^ B cells and additional B cell populations determined by standard clinical panel for lymphocyte phenotyping. (-) indicates not tested

Cell type	Percentage						Reference Range
	Patient 10.09.20	Patient 25.09.20	Patient 25.09.20	Patient 25.11.22	Mother 30.11.20	Sister 30.11.20	
CD3+ (T cells)	69.3	67.99	-	72.78	80.36	70.32	53.7-82.8
CD3+ CD4+	38.32	35.24	-	39.66	51.93	45.42	46.2-78
CD3+ CD8+	28.83	29.85	-	31.57	28.00	24.02	14.8-48.4
CD16+CD56+ (NK cells)	6.84	7.47	-	8.12	5.69	5.02	3.8-21.5
CD19 (B Cells)	0	0	-	0	13.66	24.04	3.3-32.9
CD20^+^ CD27^+^ (memory)	-	-	26.75	30.9	36.9	41.3	44-84
CD20^+^CD27^+^IgD^-^ (switched memory)	-	-	8.72	7.7	20.2	23.5	2-14
CD20^+^CD27^+^IgD^+^ (non-switched memory)	-	-	14.4	24.2	16.7	17.8	5-32
CD20^+^CD27^-^IgD^+^ (naïve B cells)	-	-	72.5	66	59	54.3	5-33
CD20+CD38^HI^CD24- (plasmablasts)	-	-	0.1	0.3	0.1	0.2	0.2-5

### A Novel Biallelic Variant in CD19 Identified by Long-Read Nanopore Sequencing

The molecular basis for the complete lack of B cell CD19 was presumed to be autosomal recessive CD19-deficiency and targeted mutation analysis of the *CD19* gene was requested.

**Fig. 2:**
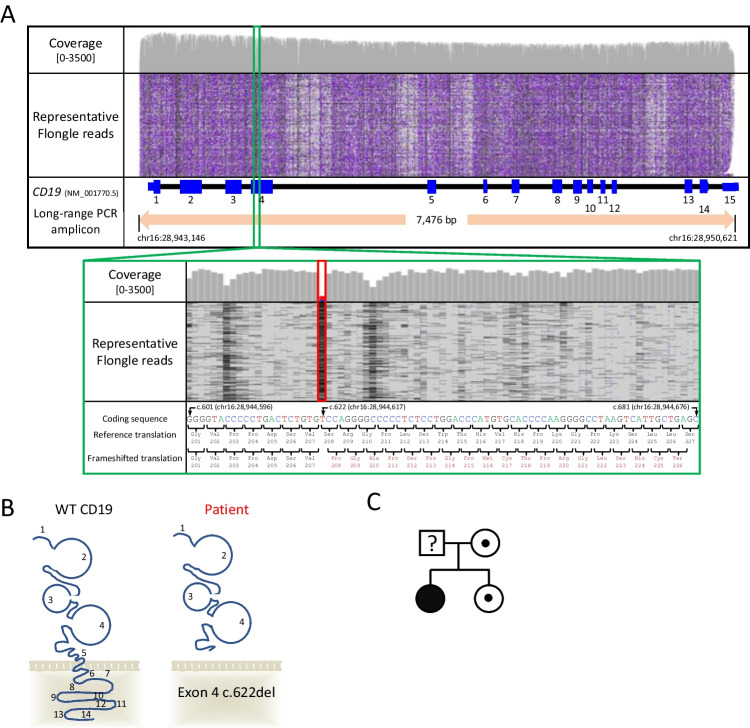
Long-read sequencing showing representative reads supporting the identification of the homozygous c.622del (NM_001770.5) (p.Ser208Profs*19) variant in *CD19* exon 4. (A) Deletion-spanning gapped alignments are depicted by black lines in alignment track. The cumulative read count is displayed per nucleotide (*y*-axis scale is 0-3500). To aid visualization the IGV’s “quick-consensus mode” was used. Transcript and genomic coordinates are provided according to NM_001770.5 and human genome build hg19 respectively. IGV: Integrative Genomics Viewer. (B) Schematic diagram of CD19 structure in cell surface with numbers indicating associated exons and predicted impact of patient variant leading to a truncated protein that terminates before the transmembrane region. (C) Pedigree of patient and unaffected family members. Affected patient with homozygous variant is indicated by filled black symbol, heterozygous family members are indicated by white symbols with a dot. The *CD19* variant status of the father was not tested

Long-read nanopore sequencing was performed on locus-specific long-range amplification products. Two possibly relevant DNA sequence variants were initially identified, and their clinical significance was interpreted according to criteria recommended by the Association for Clinical Genomic Science [Ellard et al., (2020)]. The first of these was the homozygous missense variant c.520C>G (NM_001770.5) (p.Leu174Val) which was reported at high frequency in the gnomAD control cohort (rs2904880: 0.2761) and was interpreted to be a benign sequence variant [32]. A second homozygous variant, c.622del (NM_001770.5) (p.Ser208Profs*19), was identified in *CD19* exon 4 which encodes part of the extracellular domain adjacent to the membrane and classified as pathogenic (Fig. 2A, B). The variant results in an out-of-frame translation that is predicted to result in nonsense mediated decay in accordance with best practice guidelines [33], and deficient gene expression. The variant had not been previously reported on ClinVar (https://www.ncbi.nlm.nih.gov/clinvar/), or the LOVD3 *CD19* gene-specific variant database (https://databases.lovd.nl/shared/variants/CD19). Consistent with a rare recessive inheritance pattern, there were no observations of this variant in the gnomAD v.2.1 dataset (consisting of 125,748 exome sequences) or in-house control databases.

The zygosity and segregation of the c.622del variant was confirmed by Sanger sequencing; the proband’s mother and sister were determined to be heterozygous carriers (Fig. 2C). A DNA sample from the patient’s father was not available.

### Ex Vivo Generation of Mature Plasma Cells

**Fig. 3:**
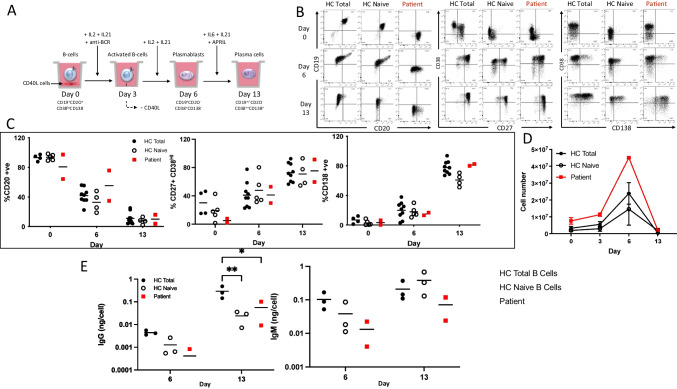
CD19-negative B cells generate plasma cells in vitro. (A) Schematic of ex vivo B cell differentiation assay. (B) Representative example of Healthy Control (HC) total B cells, naïve B cells or CD19-deficient patient B cells assessed for plasma cell generation by loss of surface CD20 and gain of CD27, CD38, CD138 after stimulation with T-dependent stimulation conditions. (C) Quantification of the percentage of cells expressing the indicated markers at the start of the assay, day 6 and day 13. HC total B cells, *n*= 9; HC naïve B cells, *n*=5; Patient, *n*=2 independent differentiations performed 5 months apart. (D) Evaluation of viable cell number during the differentiation assay. HC total B cells, *n*=4; HC naïve B cells, *n*=4; Patient, *n*=2. (E) Quantification of secreted IgM and IgG at days 6 and 13. HC total B cells, *n*=3; HC naïve B cells, *n*=3; Patient, *n*=2. BD, below detection. Significant differences were determined by two-way ANOVA (***p<0.001 **p<0.01, *p<0.05)

Previous studies have documented hypogammaglobulinemia, reduced memory B cell numbers and impaired responses to vaccination as common features of CD19-deficient patients; however, there is substantial variance in the severity of symptoms and ex vivo CD19-deficient B cell function when tested using either total peripheral blood B cells or a purified naïve population [17–21, 34, 35]. The capacity of B cells from the newly identified CD19-deficent patient to generate antibody secreting cells in response to a T-dependent like stimulation was evaluated compared to either total peripheral blood B cells or naïve-enriched, which are more similar in composition to the patient cells (Fig. 3A). When the CD19-deficient B cells were cultured under these optimized conditions, plasma cells were generated normally as assessed by loss of CD20 and gain of CD27, CD38 and CD138 (Fig. 3B,C). The CD19-deficient cells exhibited a typical pattern of initial expansion with maximal cell numbers obtained at the plasmablast stage, followed by contraction as mature plasma cells emerge (Fig. 3D). Although the levels of IgM and IgG produced in the CD19-ve cultures were relatively low at day 6, the levels became comparable to those of healthy controls derived from naïve B cells at the day 13 time point (Fig. 3E).

### Transcriptome Analysis of Differentiating Cells

**Fig. 4:**
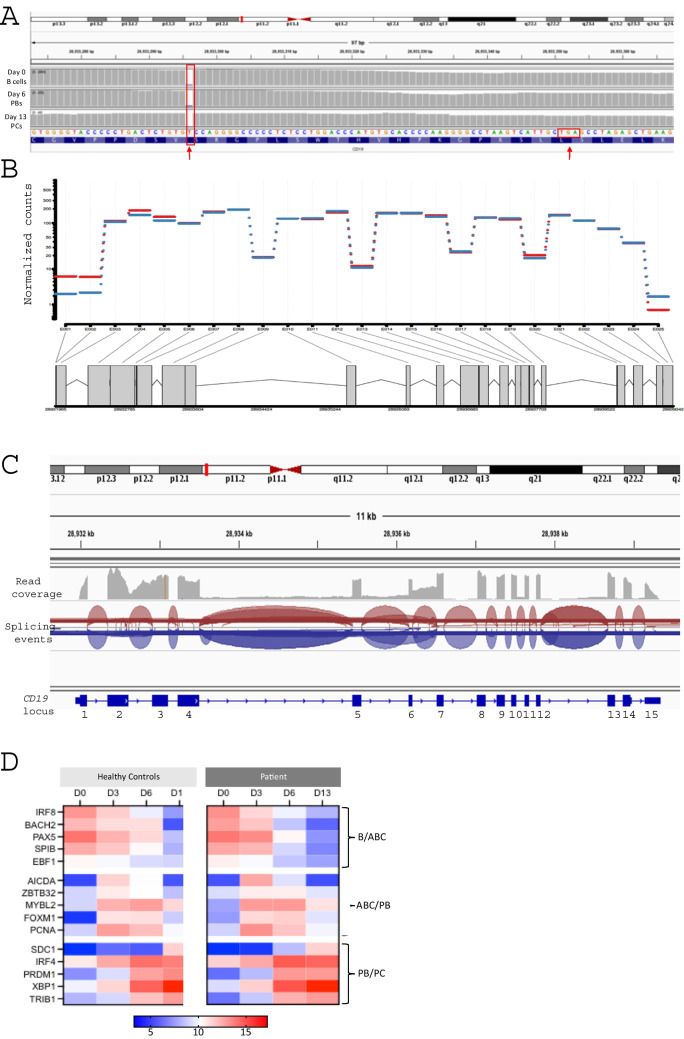
Patient B-lineage cells express *CD19* mRNA and the expected pattern of differentiation-associated genes. (A) Presence of exon 4 mutation in *CD19* reads from RNA-seq in patient differentiation samples from all time points predicted to lead to a truncated protein lacking the transmembrane domain (highlighted by arrows). (B) Relative exon usage was calculated for each exon at early (Red, days 0–3) and late (Blue, days 6–13) time points. (C) Visualization of read counts and splicing events for the *CD19* locus in day 0 B cells. (D) Heatmap of normalized expression of genes (VST) associated with B cell differentiation from heathy controls (mean expression, *n*=3) or patient samples (*n*=1). Time points (day of culture) are indicated across the top. Genes associated with B cell (B), activated B cell (ABC), plasmablast (PB) or plasma cell stages (PC) are indicated on the right

RNA sequencing was performed on material collected from cells at days 0, 3, 6, and 13, allowing for assessment of the transcriptome in resting B cells, activated B cells, plasmablasts and plasma cells, respectively, from one set of CD19-deficient samples. Analysis of the *CD19* transcripts confirmed the presence of the mutation in exon 4 in the in vitro differentiated patient samples (Fig. 4A). Despite this, investigation of the exon usage showed uniform expression of all 15 *CD19* exons and preservation of normal splicing patterns (Fig. 4B, C).

To confirm that the underlying gene expression was concordant with observed phenotypic changes, levels of key differentiation-associated genes were examined (Fig. 4D). Components of the B cell program such as *IRF8*, *BACH2*, and *PAX5* are equivalently expressed at each time point for both healthy controls and CD19-deficient cells, where expression is initially high during days 0–3 and then decreases. The CD19-deficient cells show an appropriate upregulation of the genes *AICDA* and *ZBTB32* indicative of activation at day 3, as well as several genes associated with proliferation such as *PCNA* and *FOXM1* that occurs during this phase. Additionally, genes associated with the plasma cell program are comparable, for example *IRF4*, *PRDM1*, and *XBP1* are minimally expressed before day 6, but from day 6 when the majority of the cells will be plasmablasts expression is high. Together this shows that overall differentiation control in the CD19-deficient B cells is preserved, which is consistent with the phenotype data. In depth analysis of potential differences between the healthy control and CD19-deficient samples was precluded due to the lack of replicates available from the patient.

### Signalling Events in Antibody-Secreting Cells

**Fig. 5:**
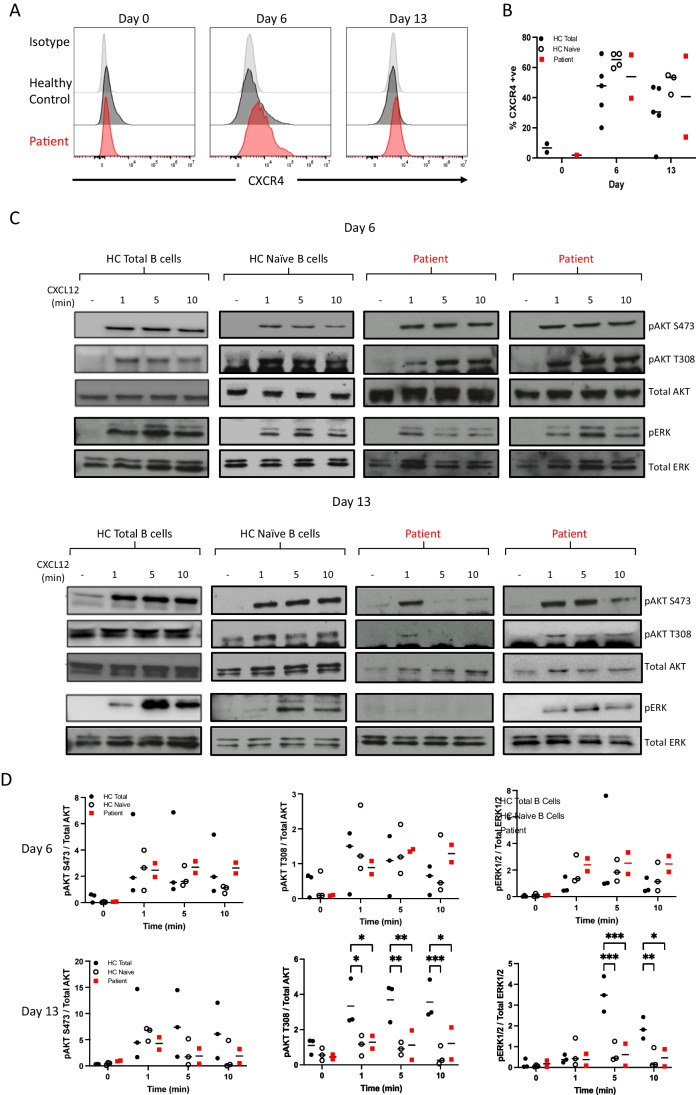
CD19-negative plasmablasts and plasma respond to CXCL12. (A) Expression of cell surface CXCR4 was evaluated on day 0 B cells, day 6 plasmablasts and day 13 plasma cells. A representative plot is shown. (B) Quantification of percentage CXCR4+ve cells in HC total B cells (*n*=5), HC naïve B cells (*n*=3), and Patient (*n*=2 independent differentiations). (C) Representative Western blot analysis of pAKT S473, pAKT T308 and pERK and total AKT or ERK protein levels from day 6 and day 13 cells stimulated with CXCL12. Both patient independent replicates are shown. (B) Quantification of relative phosphorylation from HC total B cells (*n*=3), HC naïve B cells (*n*=3), and patient samples (*n*=2 independent differentiations). Significant differences were determined by two-way ANOVA (***p<0.001 **p<0.01, *p<0.05). The images are cropped from the original full-length blots

CD19 is part of a complex that functions as a co-stimulatory element to amplify antigenic signaling via the BCR. In particular, CD19 plays a prominent role in activation of the PI3K pathway after BCR ligation. However, there is evidence that a variety of other cell surface receptors utilize CD19 to propagate PI3K signals, including the chemokine receptor CXCR4 which regulates leukocyte trafficking and survival [13]. During the in vitro differentiation, expression of CXCR4 is maximally expressed at day 6 but is also maintained on plasma cells on both healthy controls and patient cells (Fig. 4A). To determine whether CXCR4 signal propagation was affected by the loss of CD19, cells were exposed to the ligand CXCL12 and the phosphorylation of signaling intermediates was assessed (Fig. 5). In healthy controls derived from either total or naïve-enriched B cells, both day 6 plasmablasts and day 13 plasma cells showed a rapid induction of AKT and ERK phosphorylation, although the response in cells derived from a naïve starting population was relatively reduced in magnitude. This difference was most readily observed for ERK activation. The response to CXCL12 in patient plasmablasts and plasma cells was generally preserved and more in keeping with the derivation from predominantly naïve B cells. However, one replicate of the CD19-deficient plasma cells exhibited a loss ERK signaling and less extensive AKT phosphorylation.

**Fig. 6:**
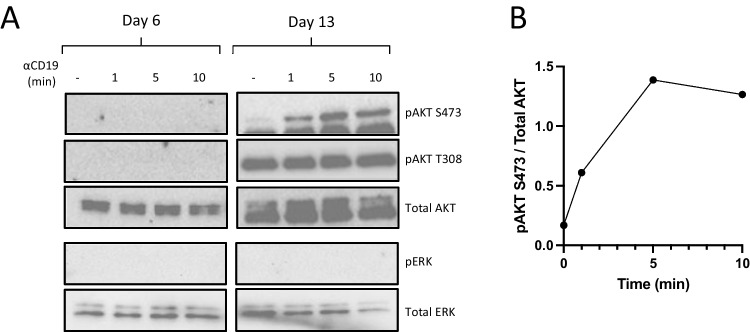
CD19 signals through AKT in plasma cells. (A) Representative Western blot analysis of pAKT S473, pAKT T308 and pERK and total AKT or ERK protein in lysates generated from day 6 and 13 cells stimulated with αCD19 microbeads for the indicated times. (B) Quantification of relative phosphorylation at day 13 from Healthy Controls (*n*=3). The images are cropped from the original full-length blots

The potentially reduced signaling in CD19-deficient plasma cells prompted us to determine whether ligation of CD19 on antibody secreting cells would result in alterations in signal transduction events as has been documented for B cells [36–40]. Day 6 plasmablasts generated from healthy control B cells were refractory to crosslinking of CD19 following exposure to anti-CD19 coupled beads (Fig. 6). In contrast, phosphorylation of pAKT S473 was detected in stimulated day 13 plasma cells. There was no evidence of changes in either pAKT T308 or phosphorylated ERK.

## Discussion

This report describes the use of third-generation sequencing methods in a patient that presented with clinical characteristics consistent with CD19-deficiency. We used nanopore sequencing to perform rapid low-cost whole-gene analysis and identified a novel homozygous frameshift mutation, c.622del (p.Ser208Profs*19), establishing a precise molecular diagnosis. The data processing pipeline we established was integrated with our standard variant annotation tools, enabling identified variants to be rapidly interpreted. Targeted genetic assays may therefore be more appropriate for patients presenting with well-defined clinical symptoms, or in whom prior investigations have revealed pathognomonic features, indicative of the underlying molecular lesion. Moreover, this experience highlights how nanopore sequencing could also be deployed as an orthogonal technology for the verification of sequence variants initially identified by short-read sequencing.

It was predicted that the introduction of a frame shift mutation seen in the CD19-deficient patient would induce nonsense-mediated decay (NMD) due to the generation of an early stop codon within exon 4. However, RNA-seq analysis indicates *CD19* expression is maintained across the entire locus. Exons 2 and 4 encode the Ig-like domains, exon 5 encodes the transmembrane region and exons 6-14 encode the cytoplasmic domains responsible for the signaling function of CD19 [41]. It is possible that the extracellular component is translated, but without the membrane spanning region and cytoplasmic tail region, the protein is not presented on the cell surface. Data from other CD19-deficient patients have also shown normal levels of *CD19* transcripts despite truncations in exons associated with the cytoplasmic domains [17]. It has therefore been suggested that the reduction in protein is due to instability of the truncated CD19 proteins. Moreover, a similar phenomenon has been observed in the context of CAR T cell therapy targeting CD19, whereby a range of mutations lead to a loss of CD19 surface expression without generating alternatively spliced variants or exhibiting NMD [42]. How these transcripts are able to avoid NMD however is unclear, though some mechanisms have been described [43].

To date, as few as 10 CD19-deficient cases have been reported in the medical literature [16]. In agreement with the other cases, the patient described herein presented with hypogammaglobulinaemia and a history of recurrent infections. Previous investigations have attributed antibody deficiency to reduced activation following antigenic stimulation, focussing on the role of CD19 in the regulation of BCR signaling thresholds. B cells from Cd19^-/-^ mice also respond poorly to mitogenic signals but can still differentiate and class switch when additional signals are provided [44]. Similarly, we found that CD19-deficient human B cells are capable of generating phenotypically mature plasma cells that secrete IgG using in vitro conditions optimized to promote maturation and viability. The phenotypic profiles were largely similar in nature, regardless of whether the starting population was derived from total B cells or a purified naïve population, and the patient samples extinguished proliferation-associated genes, suggesting that the cells had successfully progressed from plasmablasts to plasma cells. However, whether these naïve-derived cells are capable of becoming long-lived plasma cells is unknown.

Plasma cells that are destined for long-term survival are most often the product of T-dependent germinal center reactions and must localize to survival niches that provide the necessary support [45]. Such niches have been identified in a number of secondary lymphoid tissues, but the majority are located in the bone marrow [46, 47]. The ability of precursor plasmablasts to migrate to the bone marrow is dependent on the expression of chemokine receptor CXCR4 and responsiveness to its ligand CXCL12 [48–52]. The data here and previous work [53] have shown that both naïve and memory human B cells stimulated in vitro upregulate CXCR4 on the resulting plasmablasts. The in vitro generated plasmablasts from the various starting populations displayed broadly similar responses to CXCL12, suggesting that these cells are competent to home to the bone marrow. Once in the bone marrow, maintenance of PCs within the niche requires sustained contact with CXCL12 and other important survival factors such as IL-6 and BAFF/APRIL [51, 54]. Our data provides evidence that the plasma cells generated from naïve cells have reduced signaling when exposed to CXCL12 compared to those generated from total B cells, which are likely to represent outgrowth from memory B cells [30]. This may reflect the fact that the cells no longer need to remotely sense CXCL12 for migration and instead the CXCR4 signaling pathways are rewired to allow accumulation within bone marrow clusters [49]. Thus, in the CD19-deficient patient, and indeed other patients with affected memory B cell formation, the ability of plasma cells to successfully take up residence in the appropriate niches may be compromised.

CXCR4 signaling has a range of biological effects including survival, adhesion and proliferation. Its expression and downstream signaling capabilities can be modulated by co-receptors present on the cell surface [55, 56]. In B cells, there is evidence that CXCR4 works in concert with CD19 [13] and the IgD-BCR [10]. However, unlike B cells that express surface IgD, antibody secreting cells are fully capable of transmitting CXCR4 signals without the requirement for surface immunoglobulin. As CD19 acts as a central hub for relaying the downstream signal, we postulated that plasmablasts and plasma cells may continue to utilize CD19 in this capacity. Our data suggests that CD19 is not required for CXCR4 signaling in antibody secreting cells, but it is conceivable that CD19 may still play a role in amplifying low signal input as the CD19-deficient plasma cells displayed varying degrees of activation.

CD19 downregulation is consistently observed in a fraction of normal plasma cells and may relate to longevity [24, 25, 57]. However, very few differences in gene expression have been detected between CD19+ and CD19- fractions and long-term protective immunity has been documented from both types of cells [58, 59]. Our data suggests that CD19 on plasma cells does have the potential to function as a signaling element in this terminally differentiated effector population. In plasma cells, a shift in the amplitude and timing of CXCR4 and CD19 signals could lead to acute or transient changes in gene expression profiles, metabolic activity and functional behavior.

In summary, identification of a CD19-deficient patient enabled the exploration of CD19 function in late stages of B cell differentiation. While not essential for plasma cell formation, CD19 is required for signaling competence to sustain humoral immunity. Thus, the hypogammaglobulinemia observed in CD19-deficient individuals is likely to reflect a defect in memory B cell formation that subsequently impairs rapid and robust plasma cell formation to secondary exposures. This would be in line with the initial observation that vaccination with a neoantigen induces an antibody response in CD19-deficient individuals, but that the recall vaccination is impaired [17].

## Data Availability

The data that support the findings of this study are available on request from the corresponding author (SS)**.**
