# Correction to: Inherited CD19 Deficiency Does Not Impair Plasma Cell Formation or Response to CXCL12

**DOI:** 10.1007/s10875-025-01889-9

**Published:** 2025-05-29

**Authors:** Kieran Walker, Anoop Mistry, Christopher M. Watson, Fatima Nadat, Eleanor O’Callaghan, Matthew Care, Laura A. Crinnion, Gururaj Arumugakani, David T. Bonthron, Clive Carter, Gina M. Doody, Sinisa Savic

**Affiliations:** 1 Leeds Institute of Medical Research, University of Leeds, St. James’s University Hospital, Beckett Street, Leeds, LS9 7 TF UK; 2 Department of Clinical Immunology and Allergy, St James’s University Hospital, 5.18 Clinical Sciences Building, Beckett Street, Leeds, LS9 7 TF UK; 3 Yorkshire and North East Genomic Laboratory Hub, Central Lab, St. James’s University Hospital, Leeds, LS9 7 TF UK; 4 Department of Clinical Genetics, Chapel Allerton Hospital, Leeds, LS7 4SA UK; 5 National Institute for Health Research, Leeds Biomedical Research Centre and Leeds Institute of Rheumatic and Musculoskeletal Medicine (LIRMM), St James’s University Hospital, Leeds, LS9 7TF UK

**Correction to: Journal of Clinical Immunology 2023 Oct;43(7):1543–1556**

10.1007/s10875-023-01511-w

After the publication of this article, it came to our attention that Fig. 4 and Fig. 6 contained errata.

In Fig. 4, the color scale for the heatmap in part D was missing information.

In the Western blot analysis in Fig. 6A, the right panel of the figure depicting AKT at day 13 was affected by a formatting problem, leading to distortion of the image file and loss of the protein bands.

The corrected figures are shown below. We apologize for these errors, which did not affect any of the interpretations or conclusions of the article.

Figure 4D


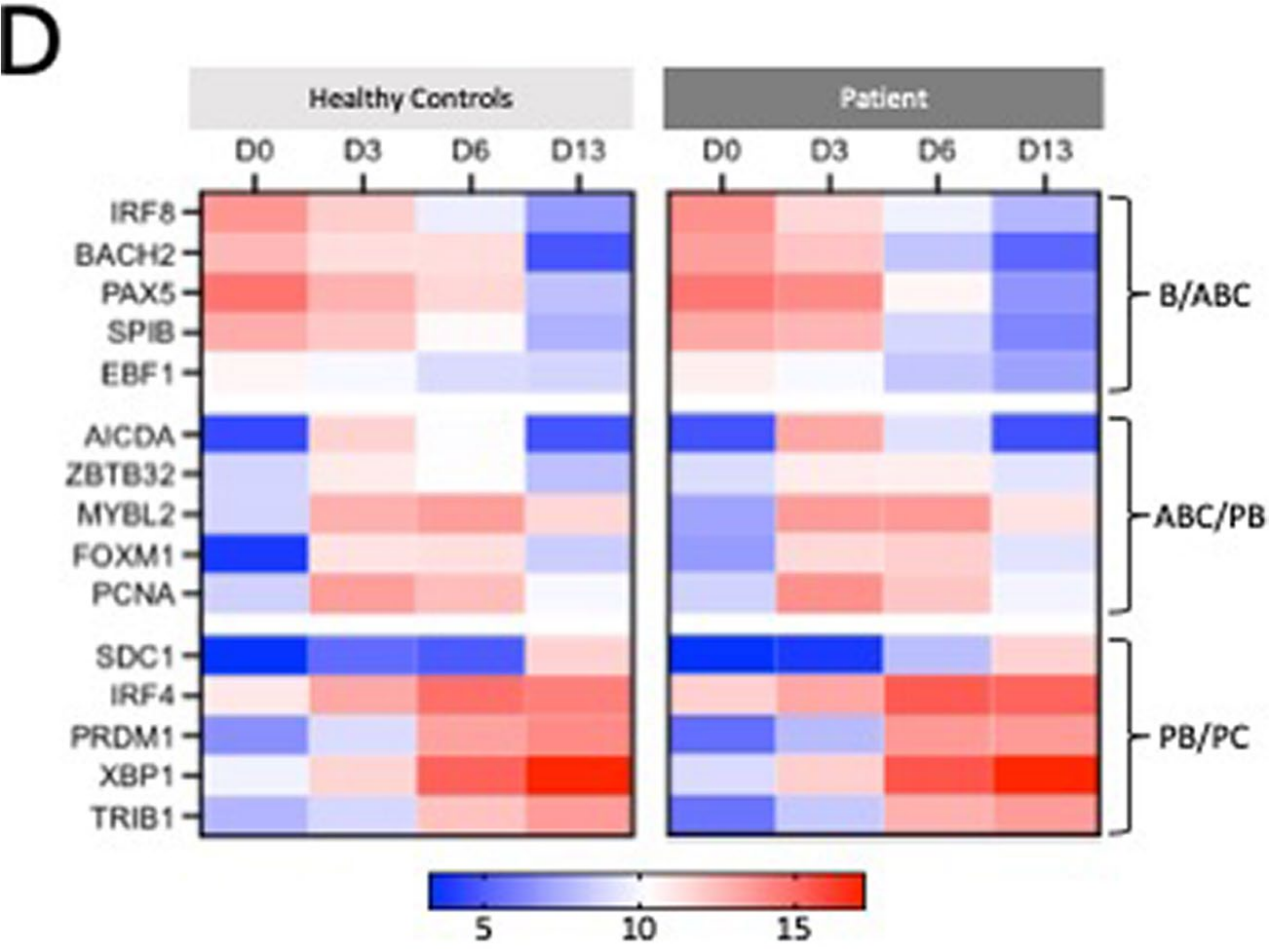


Figure 6A


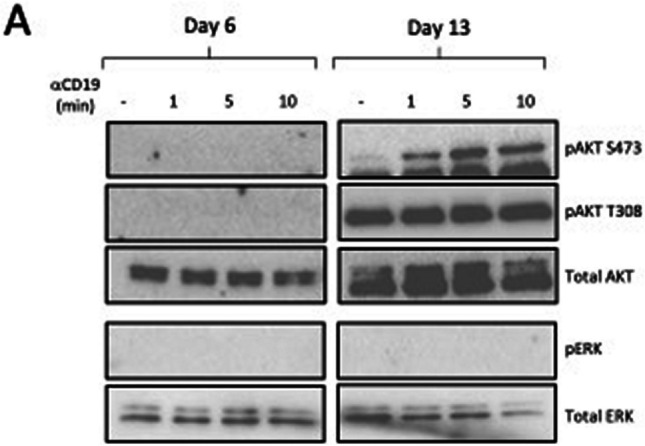


The original version has been corrected.

